# A Question

**Published:** 1801-11

**Authors:** 


To the Editors of the Medical and Physical Journal.

### A QUESTION.

Dr. Kinglake begs to know of Dr. Bouttatz, (through the medium of the Medical and Phyſical Journal) whether or not the patient who underwent the exciſion of the very extraordidinory tumour pendant from the eye, as detailed in the laſt number of the Journal,* recovered in any degree the power of viſion? In Dr. K's opinion the want of this information detracts too much both from the phyſiological and pathological value of the caſe either not to be felt by the numerous readers of Dr. Bouttatz highly intereſting narrative, or not to inſure Dr. K. an admiſſible apology for requeſting Dr. Bouttatz to ſupply what muſt have been an *accidental*, though an important omiſſion.

*Chilton super Poldon, Oct.* 12, 1801.

